# Low adherence to exclusive breastfeeding in Eastern Uganda: A community-based cross-sectional study comparing dietary recall since birth with 24-hour recall

**DOI:** 10.1186/1471-2431-7-10

**Published:** 2007-03-01

**Authors:** Ingunn Marie S Engebretsen, Henry Wamani, Charles Karamagi, Nulu Semiyaga, James Tumwine, Thorkild Tylleskär

**Affiliations:** 1 Centre for International Health, University of Bergen, Norway; 2 A2Z Project, Kampala, Uganda; 3 Department of Paediatrics and Child Health, Makerere University, Kampala, Uganda; 4 Makerere University Clinical Epidemiology Unit, Kampala, Uganda

## Abstract

**Background:**

Exclusive breastfeeding is recommended as the best feeding alternative for infants up to six months and has a protective effect against mortality and morbidity. It also seems to lower HIV-1 transmission compared to mixed feeding. We studied infant feeding practices comparing dietary recall since birth with 24-hour dietary recall.

**Methods:**

A cross-sectional survey on infant feeding practices was performed in Mbale District, Eastern Uganda in 2003 and 727 mother-infant (0–11 months) pairs were analysed. Four feeding categories were made based on WHO's definitions: 1) exclusive breastfeeding, 2) predominant breastfeeding, 3) complementary feeding and 4) replacement feeding. We analyzed when the infant fell into another feeding category for the first time. This was based on the recall since birth. Life-table analysis was made for the different feeding categories and Cox regression analysis was done to control for potential associated factors with the different practices. Prelacteal feeding practices were also addressed.

**Results:**

Breastfeeding was practiced by 99% of the mothers. Dietary recall since birth showed that 7% and 0% practiced exclusive breastfeeding by 3 and 6 months, respectively, while 30% and 3% practiced predominant breastfeeding and had not started complementary feeding at the same points in time. The difference between the 24-hour recall and the recall since birth for the introduction of complementary feeds was 46 percentage points at two months and 59 percentage points at four months. Prelacteal feeding was given to 57% of the children. High education and formal marriage were protective factors against prelacteal feeding (adjusted OR 0.5, 0.2 – 1.0 and 0.5, 0.3 – 0.8, respectively).

**Conclusion:**

Even if breastfeeding is practiced at a very high rate, the use of prelacteal feeding and early introduction of other food items is the norm. The 24-hour recall gives a higher estimate of exclusive breastfeeding and predominant breastfeeding than the recall since birth. The 24-hour recall also detected improper infant feeding practices especially in the second half year of life. The dietary recall since birth might be a feasible alternative to monitor infant feeding practices in resource-poor settings. Our study reemphasizes the need for improving infant feeding practices in Eastern Uganda.

## Background

The Millennium Development Goal 4 (MDG-4) is to reduce the under-5 mortality rates by two thirds between 1990 and 2015. The MDGs were recently evaluated, and exclusive breastfeeding for six months was considered one of the most effective interventions to achieve MDG-4 [1]. Exclusive breastfeeding, giving breast milk only and no other liquids, except drops or syrups with vitamins, mineral supplements or medicines, is superior to non-exclusive breastfeeding with a protective effect against both morbidity and mortality [2,3]. An unhygienic and unsafe environment is the main contributor to child deaths worldwide, and exclusive breastfeeding is highly protective in resource-poor settings [4,5]. The fact that HIV-1 may also be transmitted through breastfeeding has caused great uncertainty whether breastfeeding can be promoted in high HIV-1-prevalence areas. There have been fears of a so-called spillover effect on HIV-1-negative mothers or mothers of unknown HIV-1 status. The spillover effect would imply that mothers change breastfeeding behaviour, meaning that they do not breastfeed, stop breastfeeding earlier or increasingly practice mixed feeding, in other words giving complementary foods in addition to breast milk. World Health Organization (WHO) has emphasized that there should be an effort to ensure positive perceptions of and attitudes towards breastfeeding within the general population [6].

Exclusive breastfeeding seems to have a protective effect on HIV-1 transmission compared to mixed feeding. Both the HIV-1-positive population in resource-poor settings and the overall population might therefore benefit from this practice [7]. Today those working on the prevention of mother-to-child transmission of HIV (PMTCT) have to acknowledge the impact of the socio-economic conditions, HIV-1 prevalence and infant mortality rate in each local setting [8]. Overall, WHO encourages exclusive breastfeeding for the first six months of life and discourages unnecessary use of breast-milk substitutes for the part of the population who do not know their HIV-1 serostatus. Replacement feeding, meaning that the infant does not receive any breast milk in addition to replacement foods, is only recommended to HIV-1-infected mothers when it is acceptable, feasible, affordable, sustainable and safe. Only when these criteria are fulfilled is it recommended to avoid all breastfeeding. Otherwise, exclusive breastfeeding is recommended during the first months of life and should then be discontinued as soon as it is feasible. This would normally imply the same conditions as for replacement feeding from birth, that is, acceptable, feasible, affordable, sustainable and safe [6].

Uganda is considered a country with a good tradition of breastfeeding [9,10]. Uganda has an infant mortality rate (IMR) of 81 per 1000 and an under-five mortality rate (U5MR) of 140 per 1000 [11]. The prevalence rate of HIV among pregnant women is estimated to be 6.2% [12].

We set out to understand the current infant feeding practices and perceptions in Mbale District, Eastern Uganda. At the time the study was designed there was no published literature available on this topic from that area. Our aim was to improve our understanding of the actual infant feeding practices based on 24-hour dietary recall and dietary recall since birth.

## Methods

### Study site

The study was performed in Mbale District, Eastern Uganda with a total population of 720,000 and a population density of 535 per square kilometre [13]. The study was done in two of the seven counties: the urban Mbale Municipality, situated approximately 230 km from Kampala, and the rural Bungokho. Mbale Municipality is the district centre and has approximately ten percent of the district population [13]. Bungokho surrounds Mbale Municipality, and the population mainly consists of subsistence farmers. Mbale Hospital is both the District and the Regional Referral Hospital. In May 2002 the antenatal clinic at Mbale Hospital added a PMTCT component to its tasks.

### Design and sampling

Mothers of infants (0–11 months) were the primary targets as respondents, but caregiver-infant pairs were allowed for data-collection. The study was planned to be large enough to assess the prevalence of semi-exclusive breastfeeding at three months. Semi-exclusive breastfeeding is exclusive breastfeeding disregarding prelacteal feeds. The assumption was that the prevalence rate of semi-exclusive breastfeeding at three months was the same as in a previous study at a rate of approximately 50%, based on a 24-hour recall [9]. The confidence interval (CI) was set to 95%. The minimum sample size of 645 children was obtained [14].

We utilized a two-stage probability proportional-to-size cluster design [15]. Randomization was done on the village level and on the household level. Uganda Bureau of Statistics in Entebbe [13] provided us with an overview of the local administrative units. A total of 793 households were visited. There were 27 non-respondents from Mbale Municipality and 3 from Bungokho. Another 36 respondents with incomplete data and caregivers who were not mothers were excluded for analysis. This resulted in a total of 727 mother-infant pairs included for analysis. The mothers and caregivers were interviewed in their households in October and November 2003 by data collectors who were fluent in the local language Lumasaaba.

### The questionnaire

Based on the discussions of four focus groups among mothers and grandmothers in the rural and urban sites, a site-specific structured questionnaire in Lumasaaba was developed and pre-tested. Thirty-five liquids and food items were asked for in a 24-hour dietary recall. The same items were asked for in a dietary recall since birth immediately after the 24-hour recall. The 24-hour recall reflected the feeding practices from the previous morning to the morning of the interview. In the dietary recall since birth the respondents were asked if any liquid and food item had been given to the infant and, if so, when that was done for the first time. The questionnaire also included questions on socio-demographic characteristics, breastfeeding, prelacteals, siblings, immunisation status and water and sanitation.

### Data handling, definitions and analysis

The data entry was done using EpiData 3.0. Data analysis was done using SPSS 14.0.1. Prelacteal feeds were defined as any food item given within the first three days. All answers about food items were grouped in four feeding categories modified according to the WHO definition [16]: 1) exclusive breastfeeding (EBF), meaning those who had received nothing but breast milk from their mothers; 2) predominant breastfeeding (PBF), meaning those who had breast milk as their predominant source of nourishment, but with the possible addition of water and water-based drinks, fruit juice and locally made oral rehydration salts solution (ORS); 3) complementary feeding (CF), including any supplementary milk, fresh diluted and undiluted cow's and goat milk, any infant formula and milk powder or milk in tea, as well as any semi-solid and solid food with starch, fruits and vegetables, meat, fish and other protein rich products like eggs; and 4) replacement feeding (RF), including any foods or liquids except breast milk to the infant. Immunisation status was divided into adequately and not adequately immunised. For the 'adequately immunised' a one month time-lag after the national immunisation programme was allowed. Education was grouped into five categories: 1) no formal education; 2) some years of primary education, but incomplete; 3) completed primary education; 4) secondary lower education (8–10 years); and 5) secondary higher education or higher education (≥ 11 years). Marital status was divided into three categories: 1) traditional marriage, which is officially recognized in Uganda; 2) formal marriage including civil and religious marriage; and 3) other, comprising single, divorced, separated or widowed. Religion was grouped into the three main denominations: Protestant, Catholic and Muslim.

Socio-economic status was assessed by constructing an index by the use of principal components analysis (PCA). The following domains went into the model: 1) characteristics of the dwelling, including floor, walls, roof material, number of rooms per household member and toilet-status; 2) main source for lighting and cooking; 3) number of beds and ownership of the ten most wanted items (radio, television, telephone, cupboard, refrigerator, bicycle, motorcycle or scooter, car or truck and any machine for earning income); and 4) ownership of the most common animals (hens, turkeys, goats, cows and pigs). Ownership of land was kept separate. Cut-off points were given for five equal groups and quintiles representing the poorest to the least poor were used for analysis.

Separate life-table analysis was done using SPSS 'survival analysis' for the different feeding categories for both the dietary recall since birth and the 24-hour dietary recall and compared. To make it simpler for the respondents, the information about the dietary recall since birth was recorded in months, with less than four weeks counting as zero months and only completed months being used for analysis. Termination of a case in the life-table analysis was the introduction of a food item discontinuing exclusive breastfeeding and starting predominant breastfeeding, and discontinuing predominant breastfeeding and starting complementary feeding. Prelacteal feeds and 24-hour recall were controlled for in the dietary recall since birth analysis. Cox regression analysis was done to check for factors associated with the different feeding practices for the recall since birth and the 24-hour recall. The practice of giving prelacteal feeds was analyzed by using binary logistic regression. For the multivariate analysis the SPSS 'conditional backward method' was used, and removal was set to 0.2. Factors controlled for were urban/rural residence, mother's age, marital status, mother's education, religion, ownership of land, socio-economic status, gender of the infant and number of siblings. Confidence intervals (CI) reported were set to 95%.

In addition to categorizing all the food items into the four different feeding categories for the life-table analysis, the food items given to more than twenty percent of the infants are presented separately. The cumulative percentages of infants receiving these food items at different points in time were obtained. Mean and 95% CI, median and ranges were used for continuous variables, and non-parametric tests were used for categorical comparisons. The significance level was set to ≤ 0.05. The clustering effect was not controlled for as the large number of primary sampling units (111) in the study reduced the effect of it [15].

### Ethics

Approval of the study was granted by Makerere University Faculty of Medicine Ethics and Research Committee, the Uganda National Council for Science and Technology and the Regional Committee for Medical Research Ethics, Western Norway. Informed consent was obtained from all the study participants, and permission was also obtained from the local administrative units.

## Results

Among the 727 mothers the mean age was 25.4 years (range 14 to 43 years). The mean age of the infants was 5.4 months (range 0.03 to 11.96 months). The mothers had an average of 6.4 years of formal education (range 0 to 16 years), and the fathers 7.7 years (range 0 to 20 years, response rate 85%). The mothers in the urban areas tended to be younger and also have higher formal education compared to the mothers in the rural areas. They had also immunised and weighed their infants to a higher degree. The mothers living in the rural areas tended to have more children than those living in the urban areas (table 1).

### Initiation of breastfeeding and prelacteal feeding

The breastfeeding experience was nearly universal. Only one of the mothers questioned had not breastfed. Of the 726 mothers having breastfed only eight (1.1%) had stopped breastfeeding mainly due to the reported feeling of not having enough milk or the reported perception of the child not being interested. All of these eight stopped before the infant was five months, the mean and median age of stopping breastfeeding being 3.0 months. Out of the 727 mothers 718 (98.8%) practiced any breastfeeding at the interview date, 712 (97.9%) breastfed both at daytime and at night, 3 (0.4%) breastfed only at daytime and the same number only at night.

The majority initiated breastfeeding during the first day, and within the third day nearly everybody had tried breastfeeding (table 2). Prelacteal feeding was given to 57.1% of the infants within the first three days, and water based liquids were the most common (table 3). The main reason the mothers reported for giving prelacteal feeds was that they had to wait until the milk started flowing. Other reasons for giving prelacteals had to do with the baby being hungry, cleaning of the baby's throat, her own pain and exhaustion after delivery, traditions and advice from health staff.

The mothers were asked whether the baby actually needed anything except breast milk for the first three days and 252 (35%) said yes, 413 (57%) said the babies did not need anything extra and the rest did not know. Of those thinking that the baby needed prelacteals 172 (68%) actually gave it, and of those who believed the infants did not need anything 206 (50%) had actually given prelacteals (p < 0.05).

The only socio-demographic factors associated with prelacteal feeding were high education and formal marriage which remained significant as protective factors against prelacteal feeding in the adjusted analysis (adjusted OR 0.5, 0.2 – 1.0 and 0.5, 0.3 – 0.8, respectively).

### Dietary recall since birth

**Figure 1:**
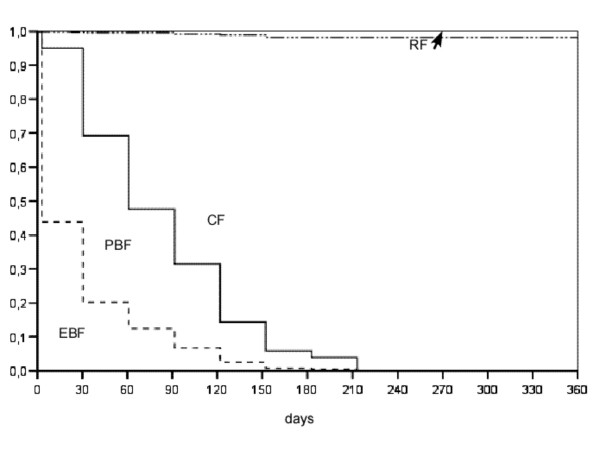
The proportion being exclusively breastfed (EBF), predominantly breastfed (PBF), complementary fed (CF) and replacement fed (RF) according to the recall since birth.

For the dietary recall since birth the proportion still practicing exclusive breastfeeding was 0.07 at three and 0.00 at six months, and the proportion still practising predominant breastfeeding was 0.30 and 0.03 at the same points in time. Figure 1 shows the life-table curves presenting the proportion discontinuing exclusive breastfeeding and starting predominant feeding, the proportion discontinuing predominant feeding and starting complementary feeding and those receiving replacement feeding, meaning not receiving any breast milk, at the different points in time. The exclusion of prelacteal feeding in the analysis yielded the same results in the life-table analysis from day 30 onwards. In the Cox regression analysis done for predominant breastfeeding (PBF) and complementary feeding (CF) no important risk factors were identified after adjustment (table 4).

### 24-hour dietary recall

The proportion who did not receive any liquids and food items in addition to breast milk, qualifying for being exclusively breastfed according to the 24-hour dietary recall, was 0.81 at three months and 0.52 at six months. This dropped steadily up to one year, but still at nine months about a quarter did not get any water or milk-based food items or semi-solid and solid food items from the previous morning to the morning of the interview.

**Figure 2:**
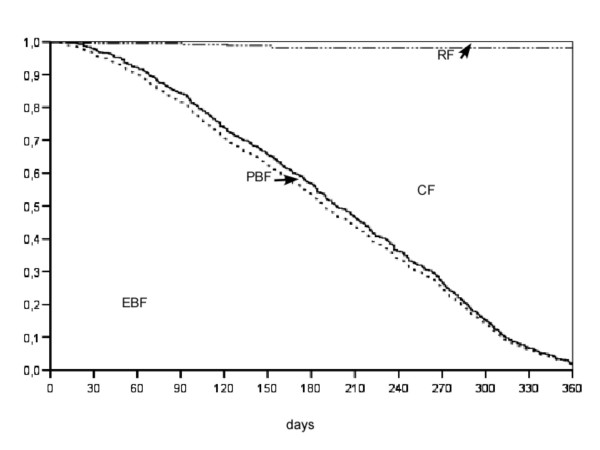
The proportion being exclusively breastfed (EBF), predominantly breastfed (PBF), complementary fed (CF) and replacement fed (RF) according to the 24-hour recall.

The life-table curve is plotted for those being exclusively breastfed, those receiving complementary feeding and those receiving replacement feeding according to the 24-hour dietary recall (figure 2). Cox regression analysis was done for both predominant breastfeeding and complementary feeding. No significant associations were seen for the predominant breastfeeding for urban/rural residence, marital status, mother's education, religion, ownership of land, socio-economic status, gender of the infant, age of infant or number of siblings. This was the same for complementary feeding except that mothers aged 25–29 tended to give complementary food items slightly less (adjusted OR 0.8, 0.6 – 0.9, p < 0.05), this was significant in both the crude and adjusted analysis.

### Comparison between the 24-hour dietary recall and the dietary recall since birth

There is a considerable difference between the 24-hour recall and the dietary recall since birth. The difference between the two methods ranges from 51 to 78% for EBF and from 30 to 59% for PBF. Table 5 sums up the cumulative percentages in the life-table analysis for both the dietary recall since birth and the 24-hour dietary recall with comparisons.

### Preferred feeding items and ages at introduction

**Figure 3:**
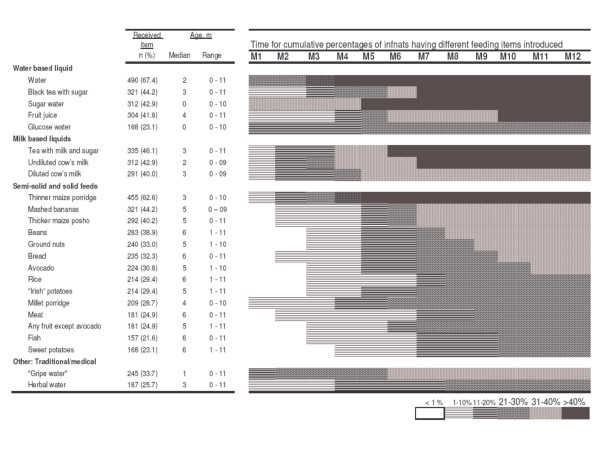
Introduction of the different food items for food items given to more than 20% of the infants, cumulative percentage. Median and range given.

Figure 3 presents the introduction of the different food items. The figure excludes liquids and food items given to less than twenty percent of the infants, such as black tea without sugar or milk, rice water, infant formula, powdered milk, goat milk, peas, cassava, sugar cane, millet bread and eggs. The figure shows that for the items being introduced, water and sugar water were introduced to the highest degree in the first month and semi-solid and solid food items, except maize porridge, in the seventh month. Milk products were mostly introduced in the fourth month together with maize porridge. It was common to give "gripe water" throughout infancy.

## Discussion

In our cross-sectional survey of 727 mother-infant pairs in Eastern Uganda in 2003, an overall picture of universal breastfeeding emerges. Despite universal breastfeeding, there is a need for improved infant feeding practices according to WHO recommendations [17]. Firstly, exclusive breastfeeding for the first six months was uncommon. This was especially obvious when 'since birth questions' were included. Secondly, frequent use of prelacteals, early introduction of many different kinds of food items, and too little complementary feeding in the second half of infancy were also seen. The feeding patterns seen in our study were not influenced by socio-demographic characteristics, which is consistent with earlier findings from Uganda and Tanzania [9,18].

In our study the majority of mothers initiated breastfeeding within the first day. The Baby Friendly Hospital Initiative (BFHI) promotes early initiation of breastfeeding, preferably within the first hour [19]. Recent findings have emphasized the risk of delayed onset of breastfeeding on neonatal mortality in a sub-Saharan setting and demonstrated that neonatal mortality could be reduced by 16% if mothers started breastfeeding at day one and 22% if they started within the first hour [20].

In our study the majority of mothers gave prelacteal feeds to the infants, a common practice among African mothers [9,10], which is discouraged by the BFHI [19]. The practice of giving prelacteals does not seem to prohibit breastfeeding from being the norm, but in our cross-sectional study possible harmful effects can not be assessed.

Infections are the estimated cause of 36% of neo-natal deaths [21]. As a post-natal intervention breastfeeding could prevent a huge amount of neo-natal deaths [22]. Culturally appropriate behavior-change communication strategies are particularly needed as an antenatal and postnatal intervention [22,23].

Studies in different settings have demonstrated different risks of too early introduction of complementary foods for the infant population (0–11 months). A multi-centre study showed increased risk of hospitalization and mortality [24]. Another Eastern European study demonstrated an increased risk of gastrointestinal tract infections and atopic eczema [25]. Promising results of exclusive breastfeeding are seen in sub-Saharan African settings, especially on reduction of HIV-1 transmission [7]. WHO encourages further studies on infant feeding practices in this latter area [16].

For the purpose of describing infant feeding practices cross-sectional surveys have been widely used [26]. There have been discussions on which recall methodology to use and how strictly to define the different feeding categories [27]. Dietary recall since birth as it is used in our study strictly emphasizes the WHO feeding definitions, focusing on the first discontinuation of exclusive breastfeeding or predominant breastfeeding [16]. Newer studies looking at HIV-1 transmission have allowed for a few lapses in exclusivity as long as it does not involve other protein-rich products like milk. There are still many uncertainties about where to put the threshold for exclusive breastfeeding and what is clinically significant when it comes to HIV-1 transmission, growth and other health outcomes [7,16]. In our study we saw that the 24-hour recall presents a picture where about half of the infant population was still exclusively breastfed after six months, compared to 0% according to the dietary recall since birth. The 24-hour dietary recall detected that about a quarter of the infants did not receive any food items from the previous morning to the morning of the interview at nine months of age. A study from Rakai, Uganda also compared the 24-hour recall with the recall since birth confirming a discrepancy in the proportion practicing exclusive breastfeeding [28]. The two recall methods describe the reality in different ways, and until we can better link any particular method with dangerous health outcomes for the infants it may be best to measure and report both ways.

Uganda Demographic and Health Survey, UDHS 2000–2001 stated that breastfeeding in Uganda is universal with 98% of children being breastfed. According to UDHS two in three children younger than six months of age are exclusively breastfed. These data are based on the 24-hour recall [29]. A review based on Demographic and Health Survey data [30] looked at breastfeeding patterns and exposure to suboptimal breastfeeding among children in developing countries and found an exclusive breastfeeding rate of 41.4% in Eastern Africa among infants up to six months. The main weakness mentioned in this review was that the survey's reported rates, particularly of exclusive breastfeeding, appeared to have a systematic upward bias, and exposure estimates should be considered conservative.

The question is whether retrospective cross-sectional methods can be a feasible alternative to prospective studies for the purpose of describing infant feeding behaviour with the methodological challenges it generates. A comparison of maternal recall methods was also done by Bland et al 2002 [31], where they compared frequent prospective 48-hour recall and seven-day recall with recall after six to nine months, and they concluded that the 48-hour recall and the six-months recall were equally poor. They recommended seven-day recall prospectively at intervals. They did not look at consecutive retrospective recalls since birth. This study was one among many which WHO's assessment tool for research from 2001 [16] was based on. The assessment tool acknowledges the complexity of infant feeding patterns as an infant can be exclusively breastfed for a period, receive other food due to a change in circumstances, and then return to exclusive breastfeeding again. This complexity can only be captured by continuous assessment which most studies will not be able to do [16]. A Swedish multi-centre longitudinal study compared the 24-hour recall with a prospective study with frequent detailed questionnaires starting 3–7 days after birth, and a difference in over 40 percentage points for EBF at age two and four months of age in the follow-up group compared to the 24-hour recall was found [32]. Likewise a Peruvian study also showed a great discrepancy between observed data, monthly reports and daily recall [33]. The discrepancy between the retrospective recall since birth and the 24-hour recall in our findings seems to be consistent with these large prospective studies.

One of the major contributions of this paper is the possibility of using 'since birth' questions in a cross-sectional survey as a complement to the 24-hour recall. The cross-sectional design is a feasible alternative compared to prospective studies, which is especially important in resource-limited settings. Given these advantages some limitations arise. The most striking limitation of the dietary recall since birth is the potential recall bias, as the mothers might forget when they introduced a food item. By recording in completed months compared to more detailed time estimation, inaccuracy is reduced at the expense of precision. According to a recent Finnish study good correlations between face-to-face recalls at three and six months and short-duration recalls by phone were shown [34]. The relative validity was especially good for breast milk and breast milk substitutes, and fairly good for other foods. Another question is if asking 'the 24-hour recall first' and thereafter 'the recall since birth' causes a certain response set. To our knowledge there are no estimates of the effect of this method. We followed a tradition of asking the most recent questions first and the least recent questions last. Similar cross-sectional surveys with a dietary recall since birth within infancy has been done earlier with face-to-face interviews and detected a similar pattern to what is found in our study [28,35,36]. A third problem which arises with face-to-face interviews is possible over-reporting of anticipated preferred behaviour [37]. In our setting with high illiteracy rates, unstable power supplies and widespread population, self-fill-in alternatives, whether paper or digital, were not feasible.

## Conclusion

Improvements in infant feeding practices are needed to reduce preventable diseases, HIV-1 transmission and mortality [1]. EBF has been emphasized in sub-Saharan Africa for two reasons: 1) it has a superior protective effect against both morbidity and mortality in the whole infant population, and 2) it generates a potentially lower postnatal HIV-1 transmission among children born to HIV-1-positive mothers. The first scientific challenge is to better understand how strictly exclusive breastfeeding needs to be in order to produce these beneficial effects. The second challenge is which methodology to use to assess exclusiveness. A 24-hour recall alone is potentially harmful as it tends to overestimate the exclusive breastfeeding practice and may give policy makers a feeling of false security so that they are not sufficiently alert to the need to focus on better breastfeeding practices. One advantage, however, is that it might detect absence of proper complementary feeding in the second half of infancy when complementary food items are recommended. The cost of the WHO suggestion of prospective frequent data collection is prohibitive for most policy purposes. Recall since birth provides a picture close to reality describing the first discontinuation of exclusive breastfeeding strictly after the WHO criteria. Combining the 24-hour recall with a recall since birth generates information that is important for policy and programme design. We therefore suggest that it is a feasible alternative to use a combination of these two methods to monitor infant feeding behavioural change interventions.

## Competing interests

The author(s) declare that they have no competing interests.

## Authors' contributions

IE was active during design, implementation, analysis and writing. HW contributed with design and co-writing. CK and NS contributed with design, implementation and co-writing. JT and TT initiated the study and contributed throughout the whole process with design, implementation, analysis and co-writing. All authors read and approved the final manuscript.

## Pre-publication history

The pre-publication history for this paper can be accessed here:



## Figures and Tables

**Table 1:** Basic socio-demographic characteristics with an urban-rural comparison.

	Urban n (%)	Rural n (%)
*Residence*	*326 (100)*	*401 (100)*
Head of household		
Male	309 (95.1)	379 (94.5)
Female	16 (4.9)	22 (5.5)
		
Mother's age		
≤19	65 (20.1)	64 (16.2)
20 – 24	126 (38.9)	121 (30.6)
25 – 29	56 (17.3)	90 (22.7)
≥30	77 (23.8)	121 (30.6)
		
Marital status		
Traditional marriage	254 (77.9)	330 (82.3)
Religious and civil marriage	39 (12.0)	39 (9.7)
Other: Single/Widowed/Separated/Divorced/No answer	33 (10.1)	32 (8.0)
		
Mother's education		
None	27 (8.3)	32 (8.0)
Stopped in primary	103 (31.6)	198 (49.4)
Completed primary	61 (18.7)	78 (19.5)
Secondary lower	67 (20.6)	66 (16.5)
Secondary higher and above	68 (20.9)	27 (6.7)
		
Father's education^a^		
None	20 (7.5)	20 (5.7)
Stopped in primary	42 (15.7)	119 (34.2)
Completed primary	55 (20.6)	99 (28.4)
Secondary lower	45 (16.9)	56 (16.1)
Secondary higher and above	105 (39.3)	54 (15.5)
		
Mother's work		
Farming	139 (42.6)	368 (92.2)
Do not farm	187 (57.4)	31 (7.8)
Additional job	84 (25.8)	49 (12.4)
No additional job	241 (74.2)	345 (87.6)
		
Father's work		
Farming	92 (30.5)	270 (71.4)
Do not farm	210 (69.5)	108 (28.6)
Additional job	265 (87.7)	235 (63.5)
No additional job	37 (12.3)	135 (36.5)
		
Religion; 3 main denominations		
Protestant	122 (37.3)	114 (28.6)
Catholic	68 (21.0)	37 (9.3)
Muslim	134 (41.4)	399 (55.2)
		
Owning land		
Yes	220 (68.1)	341 (87.0)
No	103 (31.9)	51 (13.0)
		
Socio-economic wealth index		
Bottom quintile	31 (9.6)	62 (15.5)
2^nd ^quintile	58 (18.0)	74 (18.5)
3^rd ^quintile	83 (25.8)	101 (25.3)
4^th ^quintile	75 (23.3)	75 (18.8)
Top quintile	75 (23.3)	87 (21.8)
		
Gender of infant		
Girl	156 (47.9)	190 (47.4)
Boy	170 (52.1)	211 (52.6)
		
Age of infant		
< 3 months	101 (31.0)	117 (29.2)
3 – 5 months	81 (24.8)	115 (28.7)
6 – 8 months	90 (27.6)	84 (20.9)
9 – 11 months	54 (16.6)	85 (21.2)
		
Number of siblings		
None	104 (32.0)	71 (17.8)
1	74 (22.8)	74 (18.5)
2–3	88 (27.1)	108 (27.1)
≥4	59 (18.2)	146 (36.6)
		
The infant was weighed at birth		
Yes	194 (59.7)	143 (36.0)
No	131 (40.3)	254 (64.0)
		
Owning a Child health card or any other written statement^a^		
Yes	192 (74.4)	154 (59.0)
No	66 (25.6)	107 (41.0)
		
Adequately immunised for age		
Yes	126 (38.7)	107 (26.7)
No	200 (61.3)	294 (73.3)

^a^> 10% missing values

**Table 2:** Women initiating breastfeeding at different points in time after birth.

	n (%)	n (cum. %)
Immediately	286 (39.3)	286 (39.3)
Within the first two hours	79 (10.9)	365 (50.2)
Within the first day	130 (17.9)	495 (68.1)
Within the second day	73 (10.0)	568 (78.1)
Within the third day	121 (16.6)	689 (94.7)
*Not put to the breast within the three first days*	*27 (3.7)*	

**Table 3:** Infants (n(%)) having received prelacteal feeding and the main type of prelacteals given within the first three days

	n (%)
**Not received prelacteal feeds**	**312 (42.9)**
	
**Received water based drinks**	**378 (52.0)**
Glucose water	44 (6.1)
Sugar water	156 (21.5)
Water	133 (18.3)
Salty liquid	26 (3.6)
Other kind non-milk liquid	19 (2.6)
	
**Received milk-based or semi-solid food**	**37 (5.1)**
Undiluted milk	21 (2.9)
Diluted milk	8 (1.1)
Porridge	6 (0.8)
Honey	2 (0.3)

**Table 4:** Cox regression analysis on socio-demographic factors associated with the practice of changing from exclusive breastfeeding (EBF) to predominant breastfeeding (PBF), and from PBF to complementary feeding (CF). Adjusted analysis for PBF did not give any significant results and is not presented

Variables	PBF Unadjusted		CF Unadjusted		CF Adjusted	
	OR	95%CI	OR	95%CI	OR	95%CI
Residence						
Urban	1.0		1.0			
Rural	1.1	0.9 – 1.3	1.1	1.0 – 1.3		
						
Mother's age						
≤19	1.0		1.0			
20 – 24	1.0	0.8 – 1.2	1.2	0.9 – 1.5		
25 – 29	1.0	0.8 – 1.3	1.2	0.9 – 1.5		
≥30	1.0	0.8 – 1.3	1.1	0.9 – 1.5		
						
Marital status						
Traditional marriage	1.0		1.0		1.0	
Religious and civil marriage	0.8	0.6 – 1.0	1.0	0.8 – 1.3	0.9	0.7 – 1.2
Other	1.0	0.8 – 1.3	0.7	0.5 – 0.9*	0.7	0.5 – 0.9*
						
Mother's education						
None	1.0		1.0			
Stopped in primary	1.1	0.8 – 1.4	1.1	0.8 – 1.5		
Completed primary	1.0	0.8 – 1.4	1.0	0.7 – 1.4		
Secondary lower	1.0	0.7 – 1.4	0.9	0.7 – 1.3		
Secondary higher and above	0.9	0.7 – 1.3	1.0	0.7 – 1.5		
						
Religion; 3 main denominations						
Protestant	1.0		1.0		1.0	
Catholic	0.9	0.7 – 1.1	1.1	0.9 – 1.5	1.2	0.9 – 1.5
Muslim	1.1	0.9 – 1.3	1.2	1.0 – 1.5	1.2	1.0 – 1.5*
						
Owning land						
No	1.0		1.0			
Yes	1.2	1.0 – 1.5*	1.1	0.9 – 1.4		
						
Socio-economic wealth index						
Bottom quintile	1.0		1.0			
2^nd ^quintile	0.9	0.7 – 1.2	1.1	0.8 – 1.4		
3^rd ^quintile	0.8	0.7 – 1.1	1.0	0.8 – 1.3		
4^th ^quintile	0.9	0.7 – 1.1	1.0	0.8 – 1.3		
Top quintile	0.8	0.7 – 1.1	0.9	0.7 – 1.2		
						
Gender of infant						
Boy	1.0		1.0			
Girl	1.0	0.9 – 1.2	1.0	0.8 – 1.2		
						
Number of siblings						
None	1.0		1.0			
1	1.0	0.8 – 1.3	1.3	1.1 – 1.7*		
2–3	1.1	0.9 – 1.3	1.1	0.9 – 1.4		
≥4	1.0	0.9 – 1.3	1.3	1.0 – 1.6*		

* p ≤ 0.05

**Table 5:** The table shows the percentage still practising EBF and PBF according to the dietary recall since birth and the 24-hour dietary recall at different time points. The last two columns present the difference in percentage points between the 24-hour dietary recall and the recall since birth.

Month	Dietary recall since birth		24-hour dietary recall		Difference	Difference
	EBF	PBF	EBF	PBF	EBF	PBF
1	45	68	96	98	51	30
2	12	46	90	92	78	46
3	7	30	81	84	74	54
4	3	14	70	73	67	59
5	1	6	62	65	61	59
6	0	3	52	55	52	52
7		0	42	45		45
8			33	35		
9			23	25		
10			12	13		
11			5	6		
12			0	0		

## References

[B1] Bryce J, Terreri N, Victora CG, Mason E, Daelmans B, Bhutta ZA, Bustreo F, Songane F, Salama P, Wardlaw T (2006). Countdown to 2015: tracking intervention coverage for child survival.. Lancet.

[B2] Kramer MS, Kakuma R (2004). The optimal duration of exclusive breastfeeding: a systematic review. Adv Exp Med Biol.

[B3] León-Cava N, Lutter C, Ross J, Martin L (2002). Quantifying the Benefits of Breastfeeding: A Summary of the Evidence.

[B4] Bhandari N, Bahl R, Mazumdar S, Martines J, Black RE, Bhan MK (2003). Effect of community-based promotion of exclusive breastfeeding on diarrhoeal illness and growth: a cluster randomised controlled trial. Lancet.

[B5] Arifeen S, Black RE, Antelman G, Baqui A, Caulfield L, Becker S (2001). Exclusive breastfeeding reduces acute respiratory infection and diarrhea deaths among infants in Dhaka slums. Pediatrics.

[B6] HIV and Infant Feeding Framework for Priority Action. http://www.who.int/child-adolescent-health/publications/NUTRITION/HIV_IF_Framework.htm.

[B7] Iliff PJ, Piwoz EG, Tavengwa NV, Zunguza CD, Marinda ET, Nathoo KJ, Moulton LH, Ward BJ, Humphrey JH (2005). Early exclusive breastfeeding reduces the risk of postnatal HIV-1 transmission and increases HIV-free survival. Aids.

[B8] Kuhn L, Stein Z, Susser M (2004). Preventing mother-to-child HIV transmission in the new millennium: the challenge of breast feeding. Paediatr Perinat Epidemiol.

[B9] Wamani H, Astrom AN, Peterson S, Tylleskar T, Tumwine JK (2005). Infant and young child feeding in western Uganda: knowledge, practices and socio-economic correlates. J Trop Pediatr.

[B10] Pool R, Nyanzi S, Whitworth JA (2001). Breastfeeding practices and attitudes relevant to the vertical transmission of HIV in rural south-west Uganda. Ann Trop Paediatr.

[B11] Watkins K (2005). Human Developement report.

[B12] Musinguzi J, Kirungi W, Opio A, Madraa E, Biryahwaho B, Mulumba N (2003). STD/HIV/AIDS Surveillance Report, June 2003.

[B13] Uganda Population and Housing Census 2002. Uganda Bureau of Statistics (UBOS). http://www.ubos.org.

[B14] Cochran WG (1977). Sampling Techniques.

[B15] Bennett S, Woods T, Liyanage WM, Smith DL (1991). A simplified general method for cluster-sample surveys of health in developing countries.. World Health Statistics Q.

[B16] Piwoz E (2001). Breastfeeding and replacement feeding practices in the context of mother-to-child transmission of HIV. An assessment tool for research. WHO/RHR/01.12, WHO/CAH/01.21.

[B17] Dewey KG (2001). Guiding principles for complementary feeding of the breastfed child. WHO Global Consultation on Complementary Feeding.

[B18] Shirima R, Greiner T, Kylberg E, Gebre-Medhin M (2001). Exclusive breast-feeding is rarely practised in rural and urban Morogoro, Tanzania. Public Health Nutr.

[B19] Baby Friendly Hospital Initiative (BFHI).. http://www.unicef.org/nutrition/index_24806.html.

[B20] Edmond KM, Zandoh C, Quigley MA, Amenga-Etego S, Owusu-Agyei S, Kirkwood BR (2006). Delayed breastfeeding initiation increases risk of neonatal mortality. Pediatrics.

[B21] Lawn JE, Cousens S, Zupan J (2005). 4 million neonatal deaths: When? Where? Why?. Lancet.

[B22] Darmstadt GL, Bhutta ZA, Cousens S, Adam T, Walker N, de Bernis L (2005). Evidence-based, cost-effective interventions: how many newborn babies can we save?. Lancet.

[B23] Bhutta ZA, Darmstadt GL, Hasan BS, Haws RA (2005). Community-based interventions for improving perinatal and neonatal health outcomes in developing countries: a review of the evidence. Pediatrics.

[B24] Bahl R, Frost C, Kirkwood BR, Edmond K, Martines J, Bhandari N, Arthur P (2005). Infant feeding patterns and risks of death and hospitalization in the first half of infancy: multicentre cohort study.. Bulletin of the World Health Organization.

[B25] Kramer MS, Chalmers B, Hodnett ED, Sevkovskaya Z, Dzikovich I, Shapiro S, Collet JP, Vanilovich I, Mezen I, Ducruet T, Shishko G, Zubovich V, Mknuik D, Gluchanina E, Dombrovskiy V, Ustinovitch A, Kot T, Bogdanovich N, Ovchinikova L, Helsing E (2001). Promotion of Breastfeeding Intervention Trial (PROBIT): a randomized trial in the Republic of Belarus. Jama.

[B26] Demographic and Health Surveys. http://www.measuredhs.com/aboutsurveys/dhs/manuals.cfm.

[B27] Greiner T (2002). Research on HIV and breastfeeding: definitions can make all the difference. Acta Paediatr.

[B28] Ssenyonga R, Muwonge R, Nankya I (2004). Towards a better understanding of exclusive breastfeeding in the era of HIV/AIDS: a study of prevalence and factors associated with exclusive breastfeeding from birth, in Rakai,Uganda. J Trop Pediatr.

[B29] (2001). Uganda Demographic and Health Survey 2000-2001.

[B30] Lauer JA, Betran AP, Victora CG, De Onis M, Barros AJ (2004). Breastfeeding patterns and exposure to suboptimal breastfeeding among children in developing countries: review and analysis of nationally representative surveys. BMC Med.

[B31] Bland RM, Rollins NC, Coutsoudis A, Coovadia HM (2002). Breastfeeding practices in an area of high HIV prevalence in rural South Africa. Acta Paediatr.

[B32] Aarts C, Kylberg E, Hornell A, Hofvander Y, Gebre-Medhin M, Greiner T (2000). How exclusive is exclusive breastfeeding? A comparison of data since birth with current status data. Int J Epidemiol.

[B33] Piwoz EG, Creed de Kanashiro H, Lopez de Romana G, Black RE, Brown KH (1995). Potential for misclassification of infants' usual feeding practices using 24-hour dietary assessment methods. J Nutr.

[B34] Vähätalo L, Bärlund S, Hannila ML, Uusitalo U, Pigg HM, Salonen M, Nucci A, Krischer JP, Knip M, Akerblom HK, Virtanen SM (2006). Relative validity of a dietary interview for assessing infant diet and compliance in a dietary intervention trial.. Maternal & child nutrition.

[B35] Agnarsson I, Mpello A, Gunnlaugsson G, Hofvander Y, Greiner T, Shirima R, Kylberg E, Gebre-Medhin M (2001). Infant feeding practices during the first six months of life in a rural area in Tanzania. Exclusive breast-feeding is rarely practised in rural and urban Morogoro, Tanzania. East Afr Med J.

[B36] Zhao Y, Niu AM, Xu GF, Garrett MJ, Greiner T (2003). Early infant feeding practices in Jinan City, Shandong Province, China. Asia Pac J Clin Nutr.

[B37] Waruru AK, Nduati R, Tylleskär T (2005). Audio computer-assisted self-interviewing (ACASI) may avert socially desirable responses about infant feeding in the context of HIV.. BMC medical informatics and decision making.

